# Mechanisms of Wheat Allergenicity in Mice: Comparison of Adjuvant-Free vs. Alum-Adjuvant Models

**DOI:** 10.3390/ijms21093205

**Published:** 2020-05-01

**Authors:** Yining Jin, Haoran Gao, Rick Jorgensen, Jillian Salloum, Dan Ioan Jian, Perry K.W. Ng, Venugopal Gangur

**Affiliations:** 1 Food Allergy & Immunology Laboratory, Department of Food Science and Human Nutrition, Michigan State University, East Lansing, MI 48824, USA; yining@msu.edu (Y.J.); gaohaora@msu.edu (H.G.); jorgen70@msu.edu (R.J.); salloum1@msu.edu (S.J.); jiandan@msu.edu (D.I.J.); 2 Cereal Science Laboratory, Michigan State University, East Lansing, MI 48824, USA; ngp@msu.edu

**Keywords:** wheat protein allergenicity, wheat allergy, food allergy, mouse model, adjuvant, immune markers, intrinsic allergenicity, skin sensitization, food safety, risk assessment

## Abstract

Wheat protein is considered a major type of food allergen in many countries including the USA. The mechanisms of allergenicity of wheat proteins are not well understood at present. Both adjuvant-based and adjuvant-free mouse models are reported for this food allergy. However, it is unclear whether the mechanisms underlying wheat allergenicity in these two types of models are similar or different. Therefore, we compared the molecular mechanisms in a novel adjuvant-free (AF) model vs. a conventional alum-adjuvant (AA) model of wheat allergy using salt-soluble wheat protein (SSWP). In the AF model, Balb/cJ mice were sensitized with SSWP via skin exposure. In the AA model, mice were sensitized by an intraperitoneal injection of SSWP with alum. In both models, allergic reactions were elicited using an identical protocol. Robust IgE as well as mucosal mast cell protein-1 responses were elicited similarly in both models. However, an analysis of the spleen immune markers identified strikingly different molecular activation patterns in these two models. Furthermore, a number of immune markers associated with intrinsic allergenicity were also identified in both models. Since the AF model uses skin exposure without an adjuvant, the mechanisms in the AF model may more closely simulate the human wheat allergenicity mechanisms from skin exposure in occupational settings such as in the baking industry.

## 1. Introduction

Hypersensitivity reactions to food protein allergens are commonly known as food allergies [1,2]. The prevalence of food allergy among children in the USA is 8% and among adults is 4% [1]. The prevalence of wheat allergy including sensitization to wheat proteins in the USA is 0.4% to 3.6% [3,4,5]. A similar level of prevalence is reported in Europe and Australia [6,7,8,9].

The prevalence of food allergies including wheat allergy has increased significantly during the past decade [2,10,11]. Furthermore, recent reports suggest that the severity of food-induced allergic reactions is also increasing as evidenced by potentially deadly anaphylactic reactions to food [2,10,11]. Furthermore, subjects sensitized to wheat allergens are also at an increased risk of anaphylaxis upon exercise, a condition known as exercise-induced anaphylaxis [12,13]. A recent study compared wheat food allergy prevalence in the USA among children of different racial groups. They found that it was significantly more prevalent among African Americans (23%) than among whites (7%) with food allergies (OR: 2.95). They reported that higher rates of wheat allergy might explain why there are higher rates of anaphylaxis and emergency room visits by African Americans in the USA [14]. Anaphylaxis to wheat is fairly common among the allergic subjects with more than 50% of wheat-allergic children showing anaphylaxis in one study [15,16]. Furthermore, wheat is the most common food implicated in exercise-induced anaphylaxis [13,17,18,19]. The only way to prevent such reactions is to completely avoid the exposure of the sensitive subjects to the offending food protein allergen [1,2].

Wheat is among the 8–14 major allergenic foods that are regulated for safety concerns in many countries including the USA, Canada, the European Union, Australia and New Zealand; other allergenic foods are milk, soy, egg, peanut, tree nuts, fish, mollusks, crustaceans, sesame, celery, mustard, gluten-containing cereals, lupine and sulfites [1,2,10,11,20]. Wheat is the most cultivated crop and the third most consumed cereal in the world after corn and rice [21,22]. However, wheat is the most common type of cereal grain that is linked to food allergy in many countries including the USA.

Wheat proteins are added as an ingredient to a variety of food products as well as to cosmetics [23,24,25,26]. Wheat products are also processed in a number of ways that cause changes in their protein structure/function, and potentially their allergenicity [23,27]. Furthermore, the genetic modification of wheat may also potentially alter its allergenicity [23,28,29,30,31]. Despite widespread use, changes in the allergenic potential of various types of novel and altered wheat products are largely unknown, primarily due to the lack of validated in vivo methods of allergenicity assessment [23,32]. Thus, validated in vivo methods are urgently needed to assess the intrinsic allergenic potential of altered wheat products. 

In food allergy animal studies, both adjuvant-based models as well as adjuvant-free (AF) models have been reported [23]. For wheat allergy studies, a number of animal models have been developed using rats, mice and dogs as experimental species [23,33,34,35,36,37,38,39,40,41]. Most of these models typically use adjuvants, such as alum, to enhance an allergenic response to the wheat proteins. It is reported that while the use of an adjuvant provides a convenient way to enhance the readouts of allergenicity such as IgE responses to food proteins, adjuvant-related effects may mask or exaggerate the intrinsic allergenic potential of food proteins [23]. The mechanism of how alum might enhance or mask the intrinsic allergenicity of wheat proteins is unknown at present [23,32,36,37,38,39,41,42]. Several studies suggest that a novel AF model, that uses the transdermal sensitization method, may be useful in the assessment of the intrinsic allergenic potential of food proteins [43,44,45,46,47]. Furthermore, it is unknown whether the mechanisms of wheat allergenicity are similar or different in these two different types of models. A direct side-by-side comparison of the novel AF vs. the conventional alum-adjuvant (AA) mouse models for wheat allergenicity has not been reported so far. Therefore, this study was undertaken to address these critical gaps in the science so as to advance the knowledge on the mechanisms of wheat allergenicity in mouse models. 

Here, we compared the sensitization and disease elicitation using blood markers as well as molecular immune activation signatures in the spleen of mice sensitized to salt-soluble wheat protein (SSWP) in the novel AF vs. the conventional AA mouse models. We report for the first time that the sensitization and disease elicitation responses were observed similarly in both models. However, an analysis of the spleen immune markers identified strikingly different molecular activation patterns in the spleen of these two mouse models. Furthermore, a number of molecular immune markers in the spleen associated with intrinsic allergenicity were also identified in both models.

## 2. Results

### 2.1. Comparison of Wheat Protein-Specfic IgE Antibody Responses in Adjuvant-Free vs. Alum-Adjuvant Mouse Models

**Figure 1:**
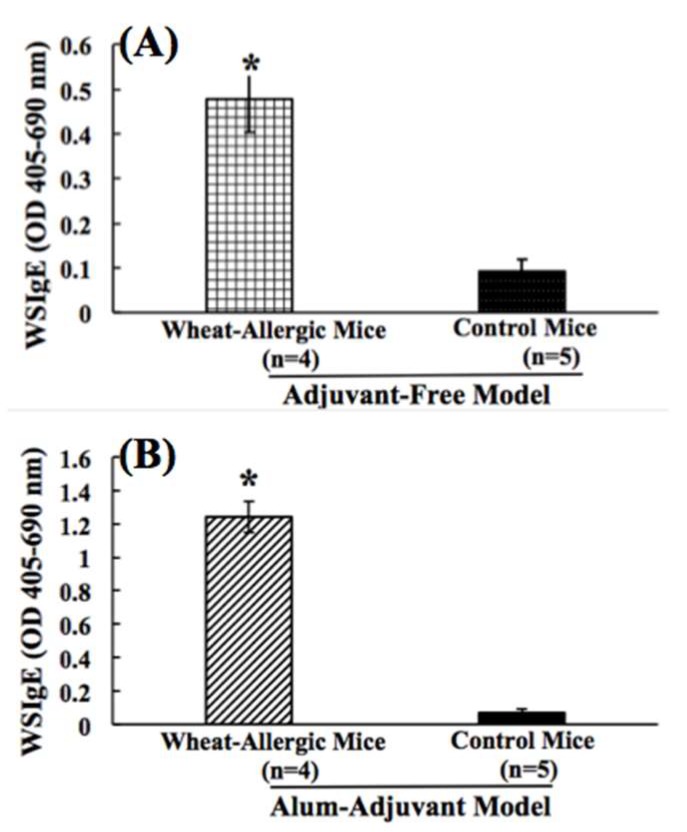
(**A**,**B**). Comparison of wheat protein-specific IgE antibody responses in the adjuvant-free (AF) vs. the alum-adjuvant (AA) mouse models of wheat allergenicity. (**A**) In the AF model, Balb/c mice were exposed to salt-soluble wheat protein (SSWP) once a week for 6 weeks via the transdermal route, as described in the methods. A group of control mice did not receive this exposure. Plasma collected after 6 weeks of exposure sensitization was used in the wheat-specific IgE (WSIgE) antibody analysis using an ELISA method described previously [32]. Figure shows the WSIgE levels in allergic mice vs. the control mice in the AF model. (**B**) In the AA model, Balb/c mice were injected with SSWP along with alum by the intraperitoneal route, as described in the methods. A group of control mice received alum only for the injection. Plasma collected after 6 weeks of sensitization was used in the WSIgE antibody analysis using an ELISA method described previously [32]. Figure shows WSIgE levels in allergic mice vs. the control mice in the AA model. * Student’s *t* test, *p* < 0.05.

The food-specific IgE antibody response is commonly used as an in vivo biomarker for allergic sensitization to food proteins [23,47]. In order to study this immune marker of wheat allergenicity, we sensitized two groups of adult Balb/c mice with SSWP extract. One group was sensitized using an AF skin sensitization method that uses six weekly applications of SSWP to the rump of mice after clipping the hair (Supplemental Figure S1A) [43,44,45,47,48,49]. Wheat-specific IgE (WSIgE) antibody levels were measured in the blood using a highly sensitive ELISA described by us [32,42]. As evident (Figure 1A), a significant elicitation of WSIgE was noted. The control group of mice did not show WSIgE responses (Figure 1A). Another group of mice was sensitized using an AA method as reported earlier (Supplemental Figure S1B) [42]. As evident (Figure 1B), a significant WSIgE response was noted. The alum-alone injected control mice did not show WSIgE responses (Figure 1B).

### 2.2. Comparison of Wheat Protein-Induced Elevation of Total Plasma IgE Antibody Levels in Adjuvant-Free vs. Alum-Adjuvant Mouse Models

**Figure 2:**
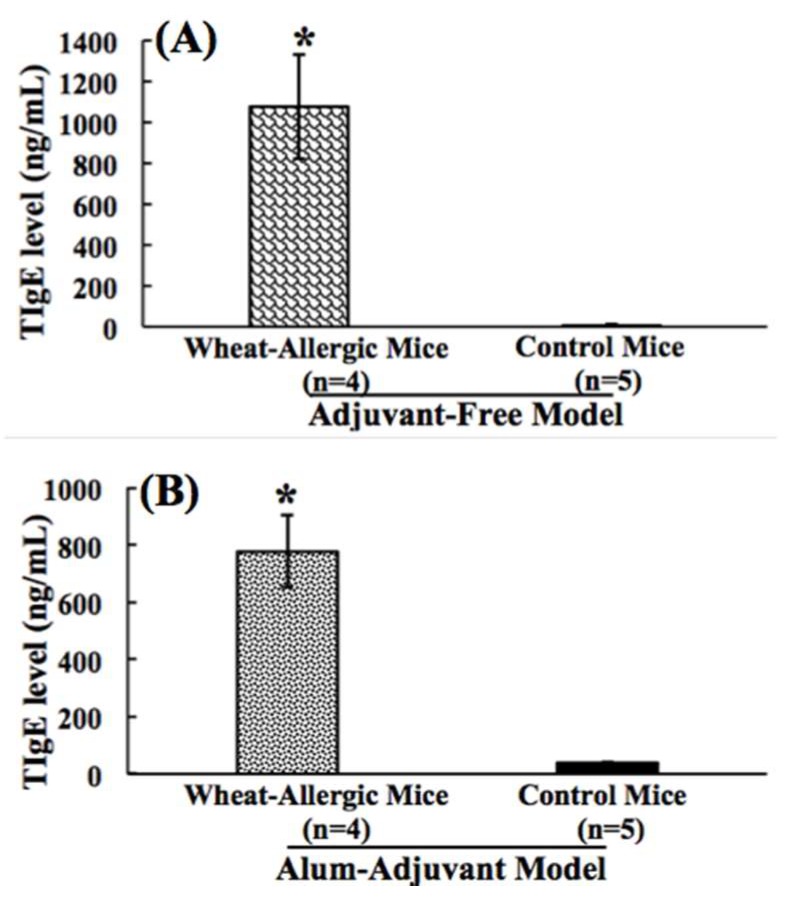
(**A**,**B**). Comparison of wheat protein-elicited plasma total IgE antibody responses in the adjuvant-free vs. the alum-adjuvant mouse models of wheat allergenicity. (**A**) In the AF model, Balb/c mice were exposed to SSWP once a week for 6 weeks via the transdermal route, as described in the methods. A group of control mice did not receive this exposure. Plasma collected after 6 weeks of exposure sensitization was used in the TIgE antibody analysis using an ELISA method described previously [42]. Figure shows the TIgE levels in allergic mice vs. the control mice in the AF model. (**B**) In the AA model, Balb/c mice were injected with SSWP along with alum by the intraperitoneal route, as described in the methods. A group of control mice received alum only for the injection. Plasma collected after 6 weeks of sensitization was used in the TIgE antibody analysis using an ELISA method described previously [32]. Figure shows the TIgE levels in allergic vs. the control mice in the AA model. * Student’s *t* test, *p* < 0.05.

An allergen-induced elevation of plasma total IgE (TIgE) levels is reported as a useful marker of allergenicity in mouse models [23,42,43,44,45,47,48,49]. Therefore, we tested this readout in this study using an optimized ELISA. In the AF model, as evident in Figure 2A, a significant elicitation of TIgE was noted. The control group of mice did not show significant TIgE responses (Figure 2A). Mice that were sensitized using the AA method also showed a significant elevation of TIgE levels (Figure 2B). The alum-alone injected control mice did not show a significant elevation of TIgE levels (Figure 2B).

### 2.3. Comparison of Wheat Protein-Specfic IgG1 Antibody Responses in Adjuvant-Free vs. Alum-Adjuvant Mouse Models

**Figure 3:**
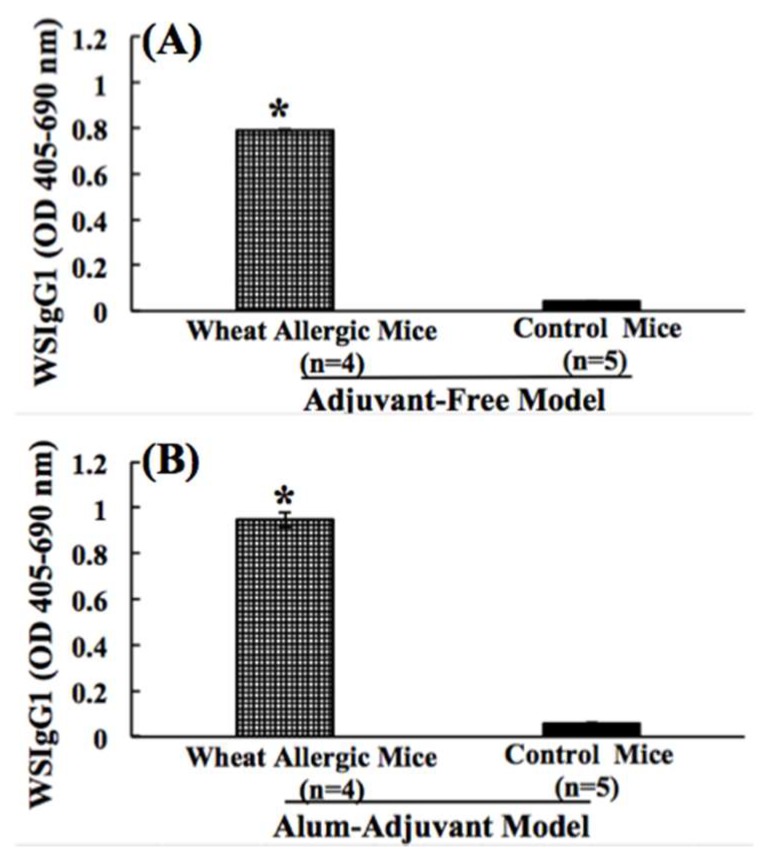
(**A**,**B**). Comparison of the wheat protein-specific IgG1 antibody responses in the adjuvant-free vs. the alum-adjuvant mouse models of wheat allergenicity. (**A**) In the AF model, Balb/c mice were exposed to SSWP once a week for 6 weeks via the transdermal route, as described in the methods. A group of control mice did not receive this exposure. Plasma collected after 6 weeks of exposure sensitization was used in the WSIgG1 antibody analysis using an ELISA method described previously [32]. Figure shows the WSIgG1 levels in allergic mice vs. the control mice in the AF model. (**B**) In the AA model, Balb/c mice were injected with SSWP along with alum by the intraperitoneal route, as described in the methods. A group of control mice received alum only for the injection. Plasma collected after 6 weeks of sensitization was used in the WSIgG1 antibody analysis using an ELISA method described previously [32]. Figure shows the WSIgG1 levels in allergic mice vs. the control mice in the AA model. * Student’s *t* test, *p* < 0.05.

In the AF model, the wheat-specific IgG1 (WSIgG1) antibody levels were measured using a highly sensitive ELISA described by us before [32,42]. As evident (Figure 3A), a significant elevation of WSIgG1 was noted in the skin-exposed mice but not in the control group (Figure 3A). In the AA model also, a significant elicitation of WSIgG1 was noted (Figure 3B). The alum-alone injected control mice did not show WSIgG1 responses (Figure 3B).

### 2.4. Comparison of Wheat Protein-Specfic IgG2a Antibody Responses in Adjuvant-Free vs. Alum-Adjuvant Mouse Models

**Figure 4:**
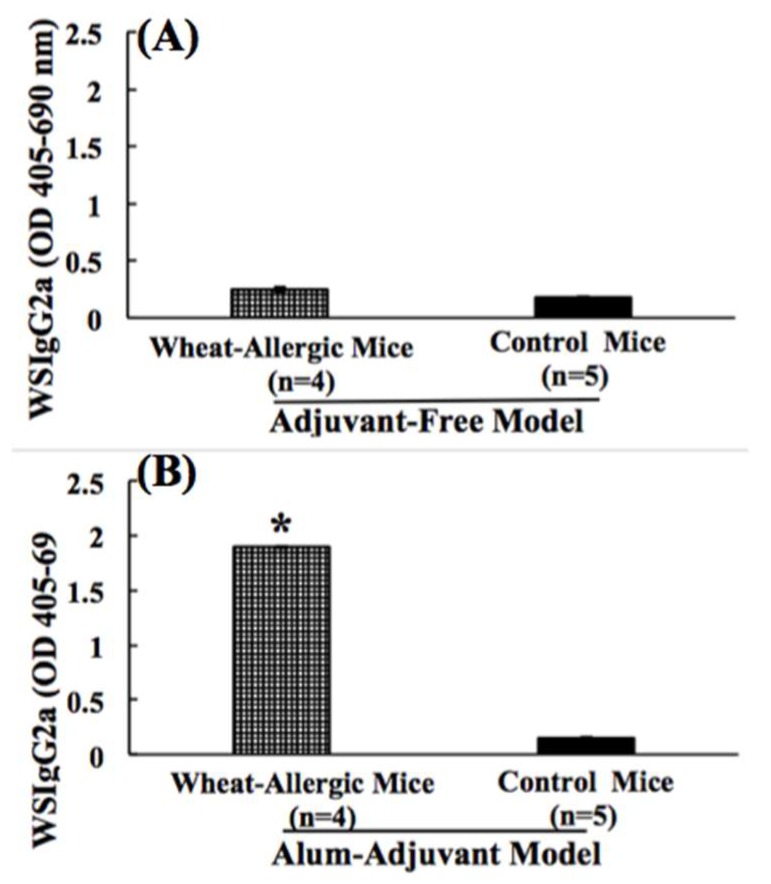
(**A**,**B**). Comparison of the wheat protein-specific IgG2a antibody responses in the adjuvant-free vs. the alum-adjuvant mouse models of wheat allergenicity. (**A**) In the AF model, Balb/c mice were exposed to SSWP once a week for 6 weeks via the transdermal route, as described in the methods. A group of control mice did not receive this exposure. Plasma collected after 6 weeks of exposure sensitization was used in the WSIgG2a antibody analysis using an ELISA method described previously [32]. Figure shows the WSIgG2a levels in allergic mice vs. the control mice in the AF model. (**B**) In the AA model, Balb/c mice were injected with SSWP along with alum by the intraperitoneal route, as described in the methods. A group of control mice received alum only for the injection. Plasma collected after 6 weeks of sensitization was used in the WSIgG2a antibody analysis using an ELISA method described previously [32]. Figure shows the WSIgG2a levels in allergic mice vs. the control mice in the AA model. * Student’s *t* test, *p* < 0.05.

The food-specific IgG2a antibody response is commonly used as an in vivo biomarker of a Th1 response because of its dependence on the Th1 cytokine IFN-g [23,47]. The wheat-specific IgG2a (WSIgG2a) antibody levels were measured in the plasma after six transdermal exposures (6R) using a highly sensitive ELISA described by us [32,42]. As evident (Figure 4A), the skin-sensitized mice did not show a marked WSIgG2a response in the AF model. However, in the AA model (Figure 4B), a significant elicitation of WSIgG2a was noted. The alum-alone injected control mice did not show WSIgG2a responses (Figure 4B).

### 2.5. Comparison of Murine Mucosal Mast Cell Protease-1 Responses in Adjuvant-Free vs. Alum-Adjuvant Mouse Models

**Figure 5:**
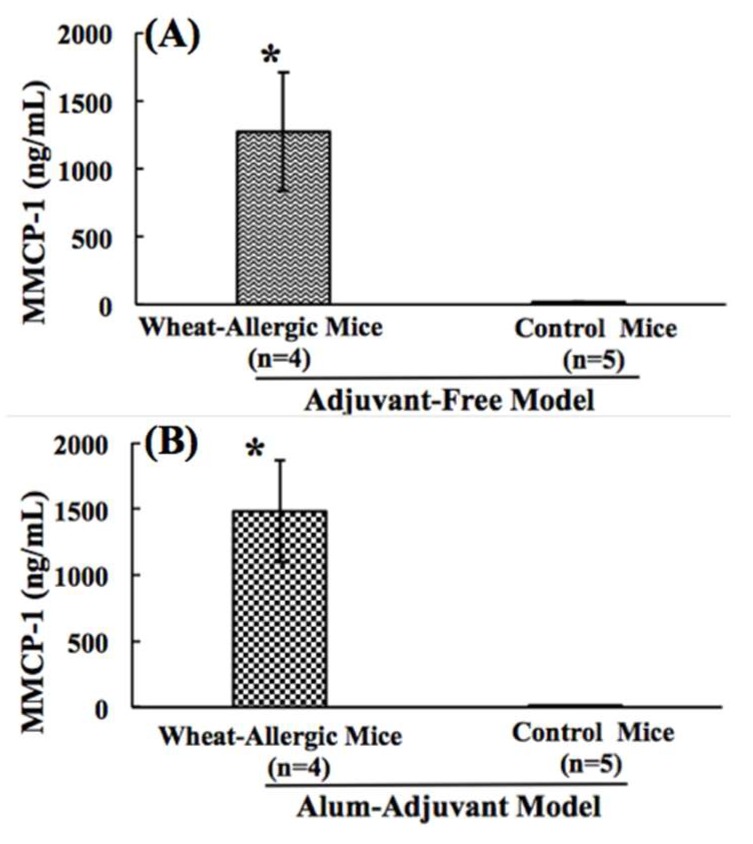
(**A**,**B**). Comparison of the mucosal mast cell protease-1 responses in the adjuvant-free vs. the alum-adjuvant mouse models. (**A**) In the AF model, Balb/c mice were exposed to SSWP once a week for 6 weeks via the transdermal route, as described in the methods. A group of control mice did not receive this exposure. Plasma collected at 1 h after the challenge with SSWP was used in the MMCP-1 protein analysis using an ELISA method described previously [42]. Figure shows the MMCP-1 levels in allergic mice vs. the control mice in the AF model. (**B**) In the AA model, Balb/c mice were injected with SSWP along with alum by the intraperitoneal route, as described in the methods. A group of control mice received alum only for the injection. Plasma collected at 1 h after the challenge with SSWP was used in the MMCP-1 protein analysis using an ELISA method described previously [42]. Figure shows the MMCP-1 levels in allergic vs. control mice in the AA model. * Student’s *t* test, *p* < 0.05.

An elevation in the plasma levels of murine mucosal mast cell protease (MMCP)-1 by 1 h after the allergen challenge is a specific biomarker of an IgE antibody-mediated anaphylactic reaction in mouse models [42,47,50]. Therefore, we compared the AF and AA models for MMCP-1 responses. As evident, in the AF model (Figure 5A), there was a significant increase in the levels of the plasma MMCP-1 protein after the SSWP challenge. In the control group of mice, there was no significant elevation of MMCP-1 protein levels (Figure 5A). In the AA model also, there was a significant increase in the levels of the plasma MMCP-1 protein after the SSWP challenge (Figure 5B). In the alum-alone injected control group of mice, there was no significant elevation of MMCP-1 protein levels (Figure 5B).

### 2.6. Analysis of in Vivo Levels of Cytokines in Adjuvant-Free vs. Alum-Adjuvant Mouse Models

We analyzed the in vivo levels of a large panel of cytokine proteins in the spleen tissue of the AF and AA models. As evident (Table 1), in the AF model, the following cytokines showed significant elevations in the allergic mice compared with the control mice: IL-4, IL-5, IL-7, IL-10, IL-12p70, IL-17B, IL-17E (IL-25), IL-17F, IL-20 and IL-23. In the AA model, the injection with alum-alone significantly increased a number of cytokines including the prototypic Th2 cytokine IL-4 (Table 2). Only the following cytokines showed a significant elevation in the allergic mice compared with the alum-alone injected control mice (Table 3): IFN-g (Th1 marker), IL-5 and IL-13 (Th2 markers), IL-2 and IL-21.

**Figure 6:**
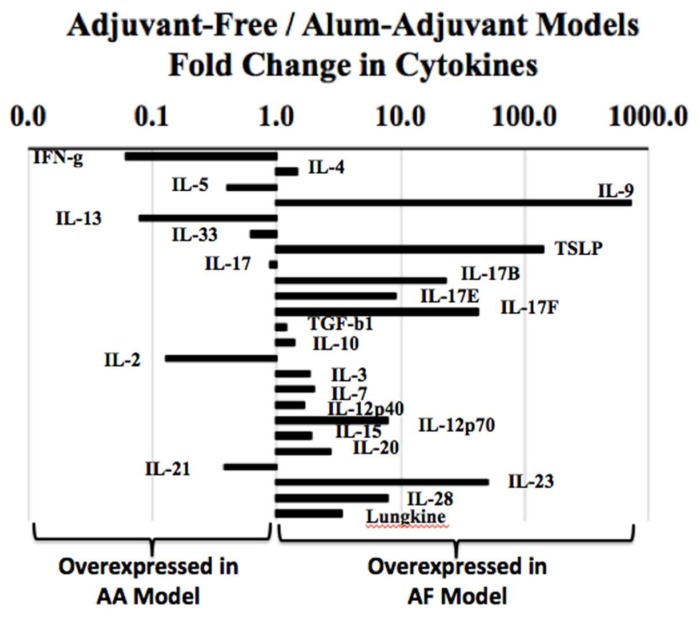
Analysis of in vivo activation of cytokines in the spleen in adjuvant-free vs. alum adjuvant mouse models of wheat allergy. Spleen tissues were collected from the experimental groups of mice in the AF vs. AA models, and used in the cytokine protein analysis, as described in the methods. Using the pg/mg spleen protein content, the fold changes in the cytokines in the AF vs. AA models were determined as shown. Data shows that different sets of cytokines are overexpressed in vivo in the AF vs. AA models, indicating the different mechanisms of wheat allergenicity in these two types of models.

Then, we compared the relative overexpression of cytokines in the AF vs. the AA model by conducting a fold-change analysis of the cytokine levels in the AF model relative to the AA model. As evident (Figure 6), different sets of cytokines were overexpressed in these two types of models indicating a marked difference in the cytokine-mediated mechanisms of wheat allergenicity in these two models.

### 2.7. Analysis of in Vivo Levels of Chemokines in Adjuvant-Free vs. Alum-Adjuvant Mouse Models

We analyzed the spleen levels of a panel of chemokine proteins. In the AF model, the following chemokines showed significant elevations in the allergic mice compared with the control mice: CXCL4 (PF-4), CXCL11 (I-TAC), CCL4 (MIP-1b), CCL5 (RANTES), CCL9 (MIP-1g), CCL11 (eotaxin), CCL19 (MIP-3b) and CCL22 (MDC) (Table 1). In the AA model, the injection with alum-alone significantly elevated a number of chemokines, thus making them inappropriate immune markers for the assessment of the intrinsic allergenicity of SSWP (Table 2). Among the useable chemokine markers, only the following were significantly elevated in the allergic mice compared with the alum-alone injected control mice (Table 3): CXCL12 (SDF-1a), CXCL13 (BLC), CCL3 (MIP-1a), CCL12 (MCP-5), CCL21 (6Ckine) and XCL1 (Lymphotatin).

**Figure 7:**
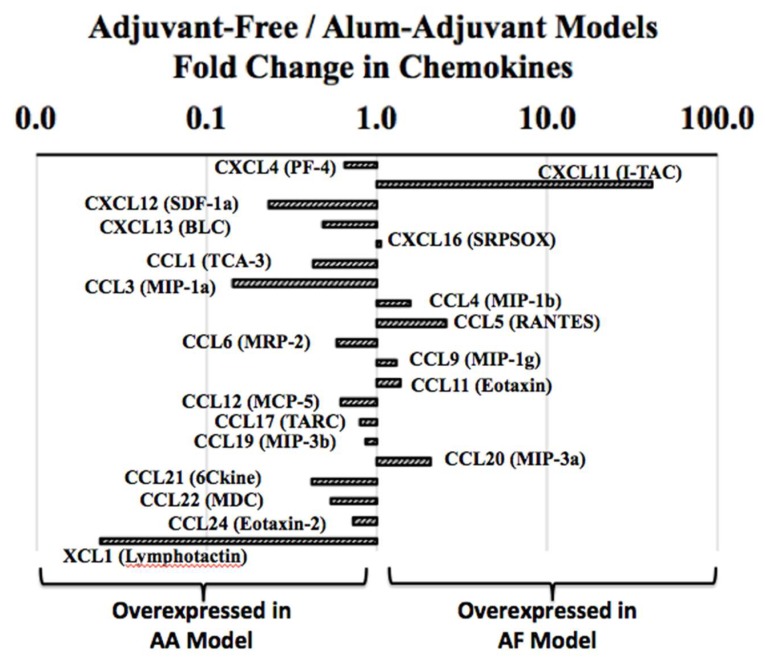
Analysis of in vivo activation of the chemokines in spleen tissue in the adjuvant-free vs. alum adjuvant mouse models of wheat allergy. Spleen tissues were collected from the experimental groups of mice in the AF vs. AA models, and used in the chemokine protein analysis, as described in the methods. Using the pg/mg spleen protein content, fold changes in chemokines in the AF vs. AA models were determined as shown. Data shows that different sets of chemokines are overexpressed in vivo in the AF vs. AA models, indicating the different mechanisms of wheat allergenicity.

Then, we compared the relative overexpression of chemokines in the AF vs. AA models by conducting a fold-change analysis of the chemokine levels in the AF model relative to the AA model. As evident (Figure 7), different sets of chemokines were overexpressed in these two types of models, indicating a marked difference in the chemokine-mediated mechanisms of wheat allergenicity.

### 2.8. Analysis of in Vivo Levels of Adhesion Molecules in Adjuvant-Free vs. Alum-Adjuvant Mouse Models

We analyzed the spleen levels of a number of adhesion molecules. In the AF model (Table 1), E-Selectin, VCAM-1, MadCAM-1, P-Cadherin and E-Cadherin were all significantly elevated in the allergic mice compared with the control mice. In the AA model, the injection with alum-alone significantly increased the levels of VCAM-1 and P-Cadherin, thus making them not useful as markers for the assessment of the intrinsic allergenicity of SSWP (Table 2). In the AA model, the allergic mice showed significant elevations of only the P-Selectin and MadCAM-1 proteins (*p* < 0.05) (Table 3).

**Figure 8:**
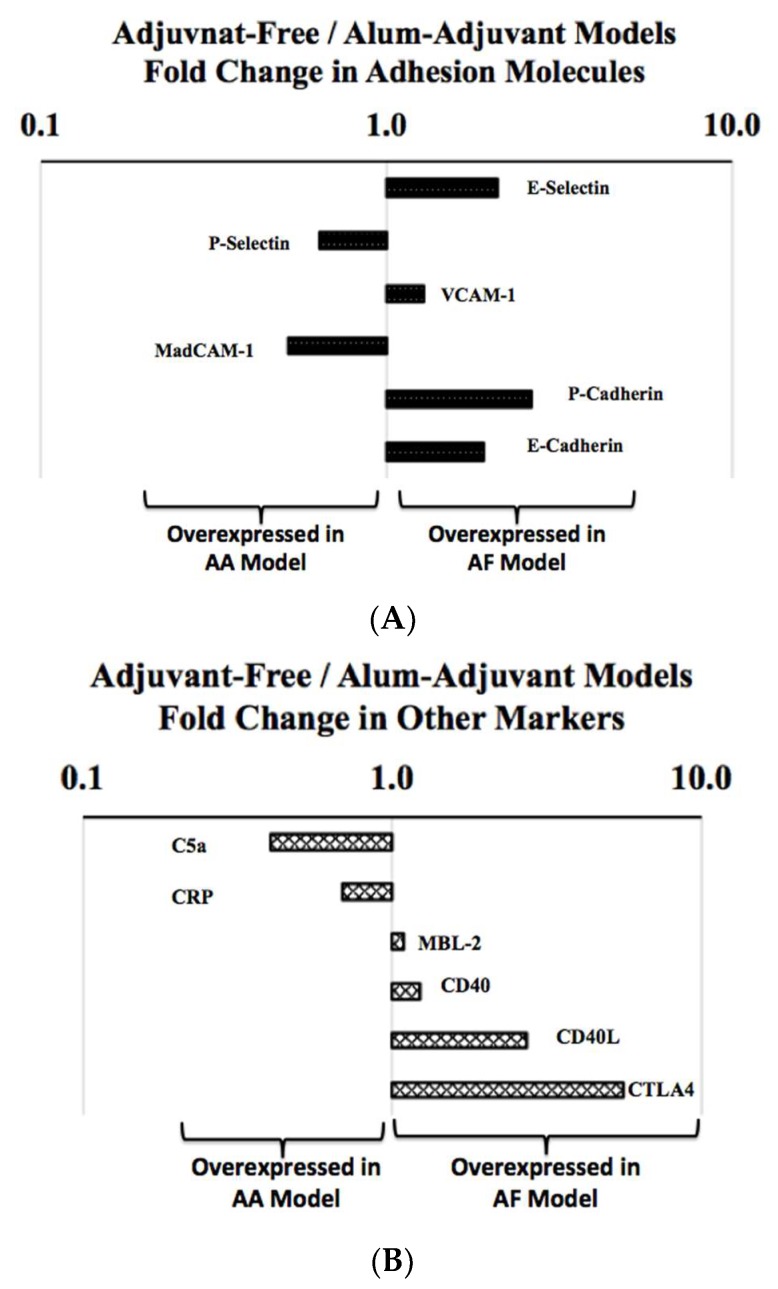
(**A**,**B**). Analysis of the in vivo activation of the adhesion molecules and other allergy-relevant immune markers in the spleen tissue of adjuvant-free vs. alum adjuvant mouse models of wheat allergy. Spleen tissues were collected from the experimental groups of mice in the AF vs. AA model, and used in the analysis of the adhesion molecules and other immune markers, as described in the methods. Using the pg/mg spleen protein content, fold changes in these markers in the AF vs. AA models were determined as shown. Data shows that the adhesion molecules (Figure **A**) and other immune markers (Figure **B**) are differently overexpressed in vivo in the AF vs. AA models, indicating the different mechanisms of wheat allergenicity.

Then, we compared the relative overexpression of adhesion molecules in the AF vs. AA models by conducting a fold-change analysis in the AF model relative to the AA model. As evident (Figure 8A), different sets of adhesion molecules were overexpressed in these two types of models, indicating a marked difference in the mechanisms of wheat allergenicity.

### 2.9. Analysis of in Vivo Levels of Other Allergenicity Relevant Immune Markers in Adjuvant-Free vs. Alum-Adjuvant Mouse Models

Finally, we evaluated a number of other immune markers relevant to allergic responses. As evident in the AF model (Table 1), significantly increased levels of C5a, CRP, CD40, CD40L and CTLA4 were noted in the allergic mice compared with the control mice. In the AA model (Table 2), alum alone significantly increased the levels of C5a, CRP and MBL-2, thus making them not useful in the analysis of the intrinsic allergenicity of SSWP (Table 2). In the AA model, the allergic mice showed significant elevations of only the CD40 and CD40L proteins (Table 3).

Then, we compared the relative overexpression of these molecules in the AF vs. AA models by conducting a fold-change analysis in the AF model relative to the AA model. As evident, we found different patterns of overexpression in these immune markers in these two types of models, indicating again, a marked difference in the mechanisms of wheat allergenicity (Figure 8B).

## 3. Discussion

In this study, we tested the hypothesis that the AF vs. the AA mouse models of wheat allergenicity would show qualitatively distinct molecular mechanisms of activation in vivo. Our data show that although both the AF and AA mouse models show similar patterns of sensitization (i.e., IgE responses) and disease elicitation reactions (i.e., MMCP-1 responses), the molecular mechanisms underlying wheat allergenicity are strikingly different in these two models. Furthermore, we also report a large number of spleen immune markers associated with intrinsic wheat allergenicity in both types of models.

We report several novel findings here: (i) this is the first study to compare side-by-side the AF and AA mouse models of wheat allergenicity; moreover, such a study has not been reported for any other allergenic foods, (ii) we show for the first time that transdermal exposure to SSWP, without an adjuvant, is capable of not only eliciting IgE responses, but also clinically sensitizing Balb/c mice for IgE-mediated systemic anaphylaxis, as evaluated by the MMCP-1 responses, (iii) the alum adjuvant alone can significantly increase a large number of in vivo immune markers including the prototypic Th2 cytokine IL-4 in Balb/c mice, (iv) in both the AF model as well as the AA model, we have identified a large number of in vivo immune markers (cytokines, chemokines, adhesion molecules and other allergenicity-relevant molecules) that may be used in the pre-clinical basic research assessment of the mechanisms of the intrinsic allergenicity of SSWP in both mouse models, and (v) the molecular mechanisms underlying wheat allergenicity in these two mouse models appear to be strikingly different.

Here, we used SSWP for testing our hypothesis. This is because, although SSWP has been shown to elicit allergic responses in humans, it has not been widely studied in animal models [16,23,47]. Furthermore, a protocol for developing the AA mouse model of wheat allergenicity using SSWP was recently published [42]. For the AF model, we used a published protocol that has been previously validated for a number of other salt-soluble food proteins including sesame, hazelnut, cashew nut, milk and shellfish [43,44,45,46,48,49,51]. However, SSWP had not been tested before in the AF mouse model of food allergenicity. Thus, the AF model of wheat allergenicity using SSWP described here is a novel mouse model.

We used SIgE and TIgE as the in vivo immune markers of sensitization to SSWP in this study. It is well known that food protein-specific IgE antibodies are necessary for initiating Type I hypersensitivity reactions, commonly known as food allergies [2]. Furthermore, these readouts have been validated for the intrinsic allergic sensitization assessment in the mouse models for a number of food proteins including peanut, egg, tree nuts, sesame, shellfish and milk [43,44,45,46,48,49,51,52,53]. Here, we show that these indicators of sensitization are significantly activated in both the AF and the AA models of wheat allergenicity.

In mice and humans, anaphylaxis can occur via both the IgG1 and IgE antibody-mediated pathways of reaction [50,54]. In this study, both IgE and IgG1 are elicited in both the AF and AA models at similar levels. Previous studies have shown that MMCP-1 is a robust and specific in vivo immune marker that distinguishes IgE antibody-mediated anaphylaxis from that of IgG1 antibody-mediated anaphylaxis in mice [47,50]. Therefore, in recent years, MMCP-1 has been validated as a highly useful in vivo immune marker of the elicitation of IgE-mediated anaphylaxis [42,47,50,54]. Here, using MMCP-1 as an in vivo immune marker, we demonstrate that in both the AF and AA models, significant IgE-mediated anaphylactic reactions occur after the SSWP challenge.

We measured IgG2a in both the AA model and the AF model in this study because in mice, the IgG2a response is a marker of the Th1 immune response as IFN-g is the prototypic Th1 cytokine that favors an antibody response to the IgG2a isotype [55,56,57]. We found that while a robust IgG2a response is noted in the AA model, a very little IgG2a response is seen in the AF model. These data correlated very nicely with the robust IFN-g response seen in the AA model and the very poor IFN-g response seen in the AF model. These data further confirm the positive relationship between IgG2a and IFN-g.

In both humans and mice, the mechanisms of the regulation of allergic immune responses are complex and involve a large number of cytokines (e.g., Th1, Th2, Th9, Th17, T regulatory cytokines, etc.), chemokines, adhesion molecules and other immune molecules [42,55,56,57]. In general, the prototypic Th1 cytokine IFN-g is considered an anti-allergic regulator, while the prototypic Th2 cytokine IL-4 is considered a pro-allergic regulator [55,56,57]. Our data, for the first time, has identified that in the AF model, as opposed to the AA model, the anti-allergic Th1 cytokine IFN-g is not significantly activated. On the contrary, the prototypic pro-allergenic cytokine IL-4 is activated by SSWP in the AF model. In contrast, in the AA model, IL-4 is activated by the alum-adjuvant alone but not by SSWP per se.

Recent studies show that the Th17 family of cytokines may play a key role in allergic responses [42,55,56]. Among these cytokines, IL-17E (also known as IL-25) is shown to be pro-allergenic, while the role of other Th17 cytokines in allergies is unclear at present [58,59]. To clarify their role in wheat allergenicity, we evaluated the panel of Th17 cytokines in this study. We found that the pro-allergenic IL-17E is activated only in the AF model but not in the AA model. In addition, we found that IL-17B and IL-17F are also activated by SSWP in the AF model but not in the AA model.

Chemokines are immune-molecules that play a key role in immune regulation, lymphocyte trafficking, inflammation and allergic diseases [56,60]. We report for the first time, that a distinct set of chemokines are overexpressed in the AF vs. the AA models. Furthermore, a number of chemokines associated with the intrinsic allergenicity of wheat protein were identified in both models. Notably, this panel includes the classical pro-allergenic CCL11 (eotaxin) and CCL5 (RANTES) chemokines that were activated only in the AF model but not in the AA model [56,57,60].

Adhesion molecules are critical players in immune cell homing to the inflamed tissue, and to lymphoid organs during immune responses, as well as in immune homeostasis during health and disease [61,62,63]. Our data shows that distinct sets of adhesion molecules are overexpressed in the AF vs. the AA mouse models of wheat allergenicity.

Acute phase response proteins such as CRP and MBL2 are the in vivo markers of systemic inflammation [64,65]. The complement-derived anaphylatoxin C5a also plays a key role in anaphylaxis [66]. Other immune markers, namely CD40 and CD40L, are critical players in the IgE class switching of B cells with a consequent promotion of allergic responses [67]. The CTLA4 molecule is an immune check point molecule that prevents the overactivation of the immune responses [68]. We found different patterns of overexpression of these molecules in vivo in the AF vs. the AA models of wheat allergenicity.

There a number of animal models reported for studying allergenicity of wheat proteins [23]. These include rat, mouse and dog species [33,36,69,70]. Here, we focused on using Balb/c mice because of their wide application in food allergenicity studies [23,38,39,40,41,43,44,45,47,48,49,70]. Bodinier et al. (2009) [70] reported ex vivo spleen IL-4/IL-5 responses to gliadins in mice. It is noteworthy that none of the previously reported animal models of wheat allergenicity conducted a comprehensive in vivo spleen immune markers analysis by including cytokines, chemokines, adhesion molecules and other immune markers as we have extensively analyzed in this study [38,39,40,41,70]. One previous study reported an immune marker analysis of skin lesions in an AA mouse model using SSWP [42]. Another study reported a limited cytokine (IL-4, IL-5, IL-10, IL-12 and GM-CSF) analysis in the lungs of mice sensitized and challenged with wheat gliadin protein in an AA mouse model [70]. Here, we show successfully for the first time that the spleen can serve as a very useful target organ for a comprehensive study of in vivo immune markers of wheat allergenicity in both the AA and the AF mouse models. Thus, we propose that an in vivo immune marker analysis of spleen tissue can be included in the assessment of the mechanisms of intrinsic wheat protein allergenicity in pre-clinical and basic research in both mouse models.

Several previous studies have used the AA model to study wheat allergenicity [38,39,40,41,70]. However, the mechanisms, as studied by spleen immune markers, had not been determined [38,39,40,41,70]. Our data now explains the underlying mechanism of how wheat protein elicits allergenicity in the AA mouse model, and thereby significantly advances the molecular mechanistic knowledge on wheat allergenicity in the AA mouse model, a significant outcome from this study. 

In our study we found that WSIgE is relatively lower in the AF model but with a higher TIgE compared with the AA model. We do not know the reason for this difference. The TIgE level includes both WSIgE and the basal total IgE that is present in the blood. In the AA model, we noted very high systemic IFN-g levels, likely due to the inflammation induced by the injection of the allergen along with alum. There was no enhanced IFN-g in the AF model. IFN-g is a strong inhibitor of total serum IgE levels [71]. We think that elevated systemic IFN-g may have reduced the basal levels of TIgE in the AA model. On the contrary, the lack of such systemic IFN-g in the AF model did not lower the basal levels of TIgE. This explains why TIgE in the AF model is higher than in the AA model, despite relatively lower WSIgE levels. Relatively higher specific IgE levels in the AA model might be due to the fact that the allergen was injected into the peritoneal cavity along with alum, which is expected to stimulate enhanced specific IgE responses. In the AA model, a transdermal allergen was applied without an adjuvant leading to relatively lower WSIgE levels.

Food allergies in general and wheat allergies in particular can be mediated by IgE or non-IgE antibody immune mechanisms [1,2,16]. An MMCP-1 analysis represents the IgE-mediated anaphylactic reactions. The Th2 cytokines such as IL-4, IL-5 and IL-13 are important for IgE responses in both humans and mice [1,2]. In non-IgE-meditated food allergies, lymphocytes, eosinophils and macrophages play an important role in the disease pathogenesis in both humans and mice [1,2,72]. These immune cells are regulated by a variety of immune modulators including cytokines, chemokines and adhesion molecules [1,2,72]. Therefore, we studied a large panel of immune markers in this model to represent both the IgE- and non-IgE-mediated regulation of wheat allergenicity.

The cytokine IL-10 is regarded as a regulatory and anti-inflammatory cytokine in humans and in some animal models. However, previous studies have shown that IL-10 is essential for Th2-mediated allergic reactions in mice when skin is used as the route for delivering the allergen [73]. In this mouse model of allergy, IL-10 is a promoter of allergic reactions. Our observations of elevated IL-10 by wheat allergens in the AF model further supports a pro-allergenic role for IL-10 in the mouse models of food allergy where allergens are administered via the skin. 

The CTLA4 molecule is an important regulator of an immune response. Previous studies of peanut allergy mouse models show that anti-CTLA4 therapy protects mice from anaphylaxis via inhibiting the mast cell responses; this suggests a pathogenic role of CTLA-4 in food allergies [74]. Our findings of elevated CTLA4 by wheat allergens in the AF model supports the previous findings of a pro-allergenic role of CTLA4 in food allergies. 

In this study, we used the spleen for the identification of in vivo molecular immune markers associated with intrinsic wheat allergenicity to be used in pre-clinical mouse model research. The use of the spleen requires euthanasia of the animals. It is noteworthy that these spleen markers are meant for use only in pre-clinical and basic research using mouse models. They are not meant for use in human patients or clinical applications. Since blood and urine are more easily available from live animals, future efforts should be placed on extending this study to include blood and urine for the identification of molecular immune markers to explain the mechanisms of wheat allergenicity.

Wheat allergy is a common type of occupational allergy reported in the baking food industry, where skin exposure to wheat protein occurs [16,17,18,19,23]. In this context, the AF model that we report here, which uses skin exposure to SSWP without an adjuvant, resembles human wheat allergenicity upon skin exposure more closely. Therefore, the mechanisms observed in the AF mouse model may more closely simulate the mechanisms of human wheat allergenicity upon skin exposure, another significant outcome from this study.

Unfortunately, at the current time, there are no published studies on cytokine and chemokine responses in human wheat allergy patients. However, we hypothesize that the findings in the AF model may be relevant to human occupational wheat allergenicity in baking industry workers, where skin exposure to wheat proteins is expected to occur. Our findings suggest that such mechanistic studies involving cytokines and chemokines are needed in human occupational wheat allergy patients with anaphylaxis.

Validated in vivo immune markers are urgently needed in the pre-clinical and basic research assessment of the intrinsic allergenic potential of food proteins in general and wheat proteins in particular. Another significant outcome of this study is that these panels of immune markers can be used in future applications in at least three areas of pre-clinical basic research to study the mechanisms of wheat allergenicity: (1) the determination of the effect of various food processing methods (e.g., thermal and non-thermal methods) on the intrinsic allergenic potential of wheat protein, (2) the determination of the effect of genetic modification by cross-hybridization or genetic engineering methods on the intrinsic allergenicity of wheat, and (3) the pre-clinical basic research testing of novel hypo/non-allergenic wheat proteins [23,32].

In conclusion, we report for the first time that (i) similar levels of sensitization and allergic reaction elicitation are observed in both the AF model and the AA model of wheat allergenicity, (ii) strikingly different activation patterns of immune molecules are observed in the spleen of these two mouse models; thus, the molecular mechanisms of wheat allergenicity appear to be different in these two types of mouse models of wheat allergenicity, and (iii) the mechanisms in the AF model, that uses skin exposure without an adjuvant, may more closely simulate the human wheat allergenicity mechanisms from skin exposure.

## 4. Materials and Methods

### 4.1. Chemicals and Reagents

Biotin conjugated rat anti-mouse IgE paired antibodies and IgE isotype standard (BD BioSciences, San Jose, CA, USA); p-nitro-phenyl phosphate (Sigma, St Louis, MO, USA); streptavidin alkaline phosphatase (Jackson ImmunoResearch, West Grove, PA, USA).

### 4.2. Mice

This study was approved by the Michigan State University Institutional Animal Care and Use Committee (IACUC ID: AMEND201900174/PROTO201900053, August 26, 2019) Weanling mice (Balb/cJ female) were purchased from The Jackson Laboratory (Bar Harbor, ME) and maintained on a plant protein-free diet (AIN-93M) throughout the study. Mice were used for sensitization when they turned 4–6-weeks old. Age- and gender- matched mice were used as the control mice and were kept in the same room. All animal procedures used were in accordance with Michigan State University policies. 

### 4.3. Preparation of Salt-Soluble Wheat Protein Extract from Durum Wheat Flour

The SSWP was prepared from durum wheat (variety Carpio) using a standard method previously published [42]. Ten grams of flour in 100 mL of 0.5 M NaCl was stirred continuously for 2 h at 20 °C followed by centrifugation (5000 g, 10 min) at 20 °C. The supernatant was frozen overnight at −70 °C and then freeze-dried. The protein content was measured by the Bio-Rad method.

*Sensitization and bleeding:* In the AF method, a group of mice (*n* = 4) were exposed to SSWP by the transdermal application (1 mg/mouse/application) once a week for 6 weeks, as described before for other food proteins (Supplemental Figure S1) [43,44,45,46,47,48]. The control group of mice (*n* = 5) did not receive SSWP but were exposed to a transdermal saline. Mice were bled before and once every two weeks after the first transdermal application with SSWP. In the AA method, a group of mice (*n* = 4) were injected via the intraperitoneal route with SSWP (0.01 mg/mouse/injection) mixed with alum (1 mg/mouse) four times: on days 0, 10, 24, and 40, as described [42]. The control mice received alum-alone as an injection. Blood was collected from the saphenous vein before exposure and after sensitization on day 46.

### 4.4. Measurement of SSWP-Specific IgE Antibody Levels and Total Plasma IgE Concentration

The WSIgE and TIgE were measured using optimized ELISA-based methods described by us previously [32,42,75]. In the TIgE ELISA, paired antibodies (i.e., a capture anti-mouse IgE antibody and a biotin-labeled anti-mouse IgE detection antibody) and an IgE isotype standard were used (BD Biosciences). In the SIgE ELISA, plates were coated with SSWP followed by blocking, sample additions, and then detection using a biotin-labeled anti-mouse IgE detection antibody (BD Biosciences), as described before [32,42].

### 4.5. Elicitation of Allergic Reaction and Quantitation of Plasma Level of Mucosal Mast Cell Protease-1

The SSWP-sensitized mice were challenged by the intraperitoneal route with SSWP (0.5 mg/mouse) (Supplemental Figure S1). The blood collected at 1 h after the challenge was used in the measurement of the MMCP-1 protein levels using an ELISA method provided by the vendor (eBioscience, San Diego, CA, USA), as described previously [42,47].

### 4.6. Spleen Tissue Collection, Protein Extraction and Analysis of Immune Markers

Mice were euthanized within 1 h after the intraperitoneal challenge with SSWP (0.5 mg/mouse). The spleen tissue from the allergic mice and from the sham-sensitized mice were collected, snap-frozen and stored in liquid nitrogen at −70 °C. The protein extract was prepared using the method described by us previously [42]. Briefly, the spleen tissue was weighed and immersed in a T-PER extraction buffer (Thermo Scientific, Waltham, MA, USA, Cat #78510) containing a protease inhibitor (Sigma-Aldrich, Saint Louis, MO, USA #P8340) (10 uL inhibitor per 1 mL of T-PER buffer). The ratio of tissue to extraction buffer (1 mL per 100 mg of tissue) was kept constant. The tissue was homogenized by ultra-sonication for 30 s, twice, at an interval of 5 min. After 15 min of resting, the mixture was centrifuged (13,500 g, 10 min) at 4 °C. The supernatant was collected and the protein concentration was determined (Bio-Rad Laboratories, Hercules, CA, USA, Protein kit). The protein supernatants were frozen in aliquots at −70 °C until sent on dry ice for the analyses of immune markers. The Ray Biotech’s Quantibody Array (RayBiotech, Atlanta, GA, USA, Mouse Cytokine Array 4000) service was used in the quantitation of the cytokines, chemokines and adhesion molecule levels. Samples were run in quadruplicate using standards as detailed by the service provider (http://www.raybiotech.com/mouse-cytokine-array-q4000.html).

### 4.7. Statistical Analysis

The comparison of the groups for significance was done using the Student’s *t*-test. An online software service was used in these analyses (http://www.socscistatistics.com/tests/). The statistical significance level was set at 0.05.

## Figures and Tables

**Table 1:** Identification of in vivo immune markers for intrinsic wheat protein allergenicity in an adjuvant-free mouse model.

Immune Markers *	Control (*n* = 5)	SSWP ** (*n* = 4)	Student’s t-Test, *p* <
***Cytokines***			
IL-4 (Th2)	0.4 ± 0.1	1.1 ± 0.1	<0.01
IL-5 (Th2)	1.3 ± 0.7	3.8 ± 0.2	<0.05
IL-7	0 ± 0	83.9 ± 27.6	<0.05
IL-10 (T-Regulatory)	89.9 ± 13.6	141.8 ± 12.2	<0.05
IL-12p70	1.3 ± 1.3	11.6 ± 2.1	<0.01
IL-20	0.7 ± 0.7	54.0 ± 13.6	<0.01
IL-23	3408.0 ± 1028.6	10,505.3 ± 2652.8	<0.01
IL-17B (Th17)	105.5 ± 4.5	219.5 ± 27.1	<0.01
IL-17E (IL-25; Th2, Th17)	54.8 ± 28.4	198.2 ± 18.4	<0.01
IL-17F (Th17)	0.3 ± 0.3	41.9 ± 9.6	<0.01
***Chemokines***			
CXCL4 (PF-4)	757.8 ± 25.9	946.8 ± 21.0	<0.05
CXCL11 (I-TAC)	13.7 ± 12.8	66.6 ± 9.8	<0.05
CCL4 (MIP1b)	10.6 ± 0.3	20.3 ± 0.9	<0.001
CCL5 (RANTES)	234.1 ± 30.4	624.1 ± 19.5	<0.0001
CCL9 (MIP-1g)	192.1 ± 19.7	289.0 ± 7.5	<0.05
CCL11 (Eotaxin)	7.2 ± 0.2	12.2 ± 0.9	<0.01
CCL19 (MIP-3b)	4.2 ± 0.4	8.4 ± 0.7	<0.01
CCL22 (MDC)	13.5 ± 0.5	19.1 ± 1.2	<0.01
***Adhesion Molecules***			
E-selectin	31.2 ± 6.9	74.7 ± 2.6	<0.01
VCAM-1	5559.8 ± 88.8	7608.9 ± 264.0	<0.001
MadCAM-1	68.7 ± 14.8	148.8 ± 25.3	<0.05
P-Cadherin	126.8 ± 36.9	378.7 ± 4.6	<0.01
E-Cadherin	465.6 ± 30.3	863.3 ± 47.1	<0.001
***Other Immune Markers***			
C5a	11.2 ± 0.7	16.3 ± 1.8	<0.05
CRP	61.6 ± 2.9	126.4 ± 14.3	<0.01
CD40	2088.4 ± 121.5	2800.4 ± 181.6	<0.05
CD40L	124.1 ± 14.6	337.5 ± 15.3	<0.0001
CTLA4	7.1 ± 1.2	18.8 ± 2.7	<0.001

* Immune markers are expressed in pg/mg of spleen protein content; ** SSWP = Salt-soluble wheat protein.

**Table 2:** Alum alone significantly increases the in vivo levels of multiple immune markers.

Immune Markers *	Control (*n* = 5)	Alum (*n* = 4)	Student’s t-Test, *p* <
***Cytokines***			
IL-1ra	72.5 ± 5.3	196.4 ± 4.1	<0.00001
IL-4 (Th2)	0.4 ± 0.1	0.8 ± 0.05	<0.01
IL-7	0	42.4 ± 9.2	<0.01
IL-9 (Th9)	100.2 ± 18.8	389.7 ± 47	<0.01
IL-12p70	1.3 ± 1.3	15.7 ± 5.5	<0.05
IL-20	0.8 ± 0.8	35.6 ± 14	<0.05
IL-28	21.7 ± 4.1	40.0 ± 5.5	<0.05
IL-33 (Th2)	156.9 ± 12.8	230.2 ± 8.2	<0.05
***Chemokines***			
CXCL4 (PF4)	757.8 ± 25.9	1030.4 ± 50	<0.01
CXCL9 (MIG)	242.9 ± 9.5	352.7 ± 9.0	<0.001
CCL1 (TCA-3)	4.6 ± 1.5	11.0 ± 2.0	<0.05
CCL9 (MIP-1g)	192.0 ± 19.7	291.4 ± 18.8	<0.01
CCL11 (Eotaxin)	7.1± 0.1	16.8 ± 0.8	<0.001
CCL22 (MDC)	13.5 ± 0.5	24.0 ± 1.6	<0.001
CCL19 (MIP-3b)	4.2 ± 0.4	6.5 ± 0.5	<0.01
***Adhesion Molecules***			
P-Cadherin	233.9 ± 36.9	428.0 ± 24.7	<0.01
VCAM-1	5559.8 ± 88.8	7046.7 ± 196.8	<0.001
***Other Immune Markers***			
C5a	11.1 ± 0.7	19.6 ± 0.4	<0.0001
CRP	61.5 ± 2.9	119.9 ± 11	<0.01
MBL-2	1485.8 ± 217.6	2117.3 ± 71.1	<0.05

* Immune markers are expressed in pg/mg of spleen protein content.

**Table 3:** Identification of in vivo immune markers for intrinsic wheat protein allergenicity in an alum-adjuvant mouse model.

Immune Markers *	Alum (n = 5)	Alum + SSWP ** (n = 4)	Student’s t-Test, *p* <
***Cytokines***			
IFN-g (Th1)	0.7 ± 0.5	42.1 ± 4.6	<0.001
IL-2	0.1 ± 0.1	27.7 ± 4.0	<0.0001
IL-5 (Th2)	0.9 ± 0.6	9.5 ± 0.6	<0.0001
IL-13 (Th2)	0.4 ± 0.4	25.9 ± 4.6	<0.01
IL-21	0.1 ± 0.1	15.8 ± 4.8	<0.05
***Chemokines***			
CXCL12 (SDF-1a)	1.7 ± 0.03	8.8 ± 0.6	<0.0001
CXCL13 (BLC)	159.9 ± 20.4	304.2 ± 13.5	<0.01
CCL3 (MIP1a)	3.6 ± 1.5	41.1 ± 4.4	<0.001
CCL12 (MCP-5)	0.9 ± 0.4	4.7 ± 1.1	<0.05
CCL21 (6Ckine)	11.1 ± 1.7	71.6 ± 16.3	<0.05
XCL1 (Lymphotactin)	69.4 ± 16.7	433.5 ± 94	<0.01
***Adhesion Molecules***			
P-selectin	14,861.9 ± 344.5	23,402.1 ± 380.6	<0.01
MadCAM-1	12.2 ± 3.5	286.2 ± 14.6	<0.00001
***Other Immune Markers***			
CD40	1829.92 ± 88.1	2274.11 ± 138.1	<0.05
CD40L	57.9 ± 5.7	124.6 ± 13.6	<0.01

* Immune markers are expressed in pg/mg of spleen protein content; * SSWP = Salt-soluble wheat protein.

## Supplementary Materials

The following are available online at https://www.mdpi.com/1422-0067/21/9/3205/s1.
