# Supplementary material for: Mechanisms of Wheat Allergenicity in Mice: Comparison of Adjuvant-Free vs. Alum-Adjuvant Models
Source: Int J Mol Sci. 2020 May 1;21(9):3205. doi: 10.3390/ijms21093205 (PMC7247356; doi:10.3390/ijms21093205)
Supplement: Supplementary file 1 [file ijms-21-03205-s001.pdf]

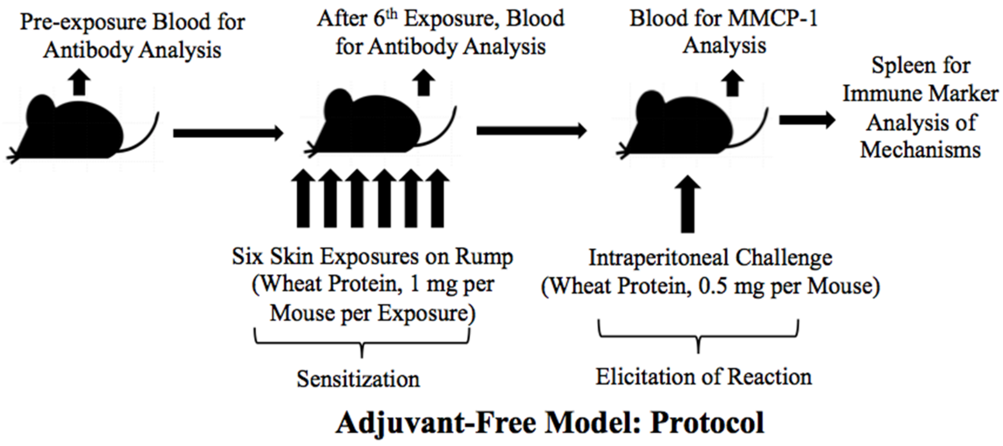

(**A**)

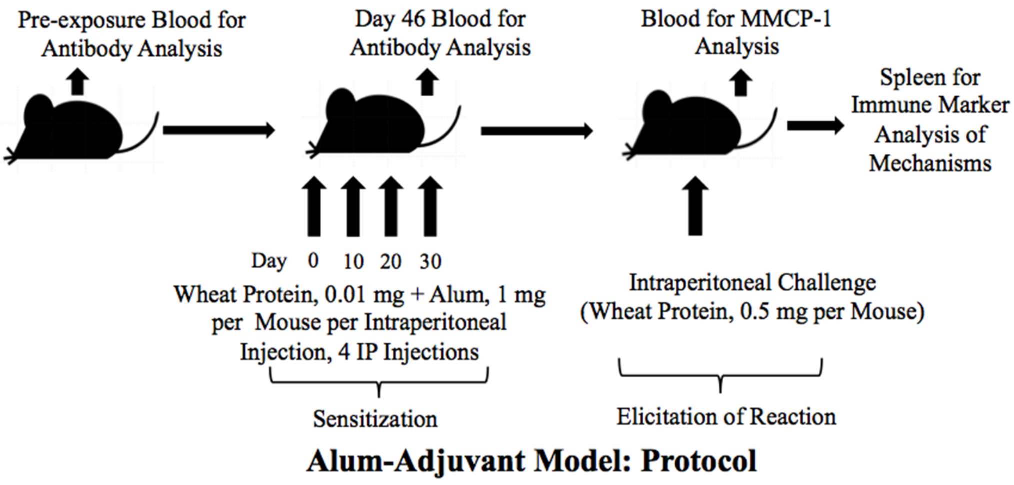

(**B**)

Supplemental Figure S1. (**A–B**). Protocols used in the adjuvant-free mouse model and the alum-adjuvant mouse model. Groups of Balb/c female adult mice were produced and maintained on a plant protein-free diet during the entire experiment. Mice were pre-bled to collect pre-exposure blood samples to measure antibodies. Figure A: shows the sensitization protocol used in the AF model. This consisted of six weekly exposures to wheat protein by the application of the protein over the intact skin on the rump. Blood collected after the 6th exposure was used in the antibody analysis. To elicit an allergic reaction, mice were challenged with wheat protein by an intraperitoneal injection. Blood collected at 1 h was used in the MMCP-1 analysis. Mice were euthanized and their spleens were collected and used in the spleen immune marker analysis. Figure B: shows the sensitization protocol used in the AA model. This consisted of 4 intraperitoneal injections of wheat protein plus an alum adjuvant. Blood collected before exposure and on day 46 after exposure was used in the antibody analysis. The elicitation of an allergic reaction and collection of samples was identical to that of the AF model. The difference between the two models is only in the sensitization protocol. The method used in the elicitation of an allergic reaction is identical in both models.
